# Therapeutic Notes

**Published:** 1896-02-15

**Authors:** 


### THERAPEUTIC NOTES.

### STYPTICEN—A NEW HÆMOSTATIC FOR GYNÆCOLOGICAL USE.

Within the last few years alkaloids, prepared from the rhizome of *Hydrastis Canadensis*, have met with some success in the treatment of uterine hæmorrhage. Hydrastinæ hydrochloras is reported to possess an ecbolic action, and hydrastinine,[1] an oxidation product of the foregoing, is undoubtedly a hæmostatic of considerable value in cases of metrorrhagia. We have now to record the introduction of a new drug—stypticen[2] by name—which, both chemically and physiologically, bears a close relationship to hydrastinine. The chemical relationship, as far as the formula is concerned, may be observed by comparing the two bodies:

$C_{11}\,H_{13}\,No_3$ = Hydrastinine

$C_{11}\,H_{12}\,No_3\,(O,\,CH_3)$ = Cotarnine (the hydro-chlorate of this base = stypticen).

The physiological or therapeutic action appears to be one of vaso-constriction, especially of the small arterioles. According to present knowledge, there is no special action on the smooth muscle fibres of the uterus, and its value in cases of uterine hæmorrhage cannot depend largely on the production of independent or active contraction of this organ.

The base cotarnine is by no means a new discovery. Many years ago the chemist Wöhler prepared it from narcotine, one of the alkaloids of opium, by treatment with oxidising agents in the presence of acids. The hydrochlorate of this base has, however, only recently been employed as a therapeutic agent, under the name of stypticen. Dr. Martin Freund suspecting, from its similarity to hydrastinine, that this body might have valuable styptic properties, entrusted its clinical investigation to Dr. Gottchalk, of Berlin. This gynæcologist, after a long series of practical experiments both on the lower animals and in female patients, has lately published an account of his researches in the pages of the "Therapeutische Monatshefte" for December, 1895. His list of cases includes examples of almost every variety of uterine hæmorrhage. Those, however, in which stypticen met with most success comprised cases of congestive dysmenorrhœa, which were complicated by excessive loss of blood. The drug appeared most efficacious when the preliminary prcaution was taken of administering it in small doses some days before the menstrual event was due. Hæmorrhage accompanying subinvolution of the uterus was successfully controlled by the exhibition of stypticen, although Dr. Gottchalk warns us that it has no noticeable influence on the active contraction of that organ; in such cases ergot should always be prescribed in combination. It appears that in cases of uterine polypi, adenoids, or other growths of the uterus, the influence of stypticen is of doubtful benefit, and more active interference is indicated.

This new hæmostatic is regarded by Dr. Gottchalk as possessing certain sedative and soothing properties of special value in dysmenorrhœa, properties which, according to his view, might reasonably be expected owing to its derivation from narcotine. This feature in its possible applications requires further corroboration, since, according to many English authorities, no narcotic properties are possessed by narcotine itself. With regard to dosage and mode of administration, when rapid effects are desired it may be given in doses of 2 grms.; ordinarily, doses of ·05 grms. every four hours are sufficient. Hypodermically, its action is rapid, and it may be injected in 10 per cent. solution into the gluteal muscles with no inflammatory effects. By the mouth it may be taken in the form of powders, pills, or perles.

[1] Lancet, 1890, i., 712; 1892, ii., 1,350; Therapeut. Gaz., 1890, 86; 1892, 539, 699. [2] Prepared by E. Merck, Darmstadt.

